# Two-Stage Management of Early Intrapelvic Cup Migration

**DOI:** 10.1016/j.artd.2026.102115

**Published:** 2026-08-14

**Authors:** Joe Ghanimeh, Kaissar Yammine, Joeffroy Otayek, Anthony El Alam, Fadi Hayek, Camille Samaha, Chahine Assi

**Affiliations:** a Department of Orthopedic Surgery, Lebanese American University Medical Center-Rizk Hospital, Lebanese American University, School of Medicine, Beirut, Lebanon; b Department of Vascular and Endovascular Surgery, Lebanese American University Medical Center - Rizk Hospital, Beirut, Lebanon

**Keywords:** Total hip arthroplasty revision, Intrapelvic cup migration, Acetabular reconstruction, Kerboull cross plate, Case report

## Abstract

Intrapelvic migration of the acetabular component following hip arthroplasty is a rare but challenging complication with significant risks, including adjacent organs and vascular injury. In the current literature, managing this complication has focused on removing the migrated failed implant first, followed by revision of the prosthesis. We present the case of a patient who sustained an early intrapelvic migration of the acetabular cup and screws following total hip arthroplasty. A unique two-stage approach was employed: acetabular reconstruction using bone graft, a Kerboull cross-plate, and a dual-mobility cup was performed first; the migrated cup and screws were safely removed subsequently through a retroperitoneal approach. The described technique highlights the efficacy of a staged surgical intervention, starting with the acetabular reconstruction, followed by delayed removal of the retained intrapelvic implant.

## Introduction

Immediate intrapelvic migration of the acetabular cup following total hip arthroplasty (THA) is a rare complication that comes with multiples challenges and life-threatening risks. Those risks include vascular injury, compression of the lumbosacral plexus, and perforation of the digestive tract, or urogenital organs [1] by the cup itself, or by its screws and pegs. This paper presents a case study that highlights the complexity of this phenomenon and introduces a unique two-stage surgical technique designed to address specific challenges associated with this complication. The first step was the THA revision with acetabular reconstruction using a Kerboull cross-plate [2] and keeping the migrated cup inside the pelvis. The second step consisted of the removal of the retained cup and screws via a retroperitoneal approach. Emphasis is placed on mitigating vascular and visceral risks, as well as overcoming difficulties encountered during the extraction of the acetabular implant.

## Case history

An 80-year-old female presented to our institution for continuous left severe groin pain and limping.

**Figure 1:**
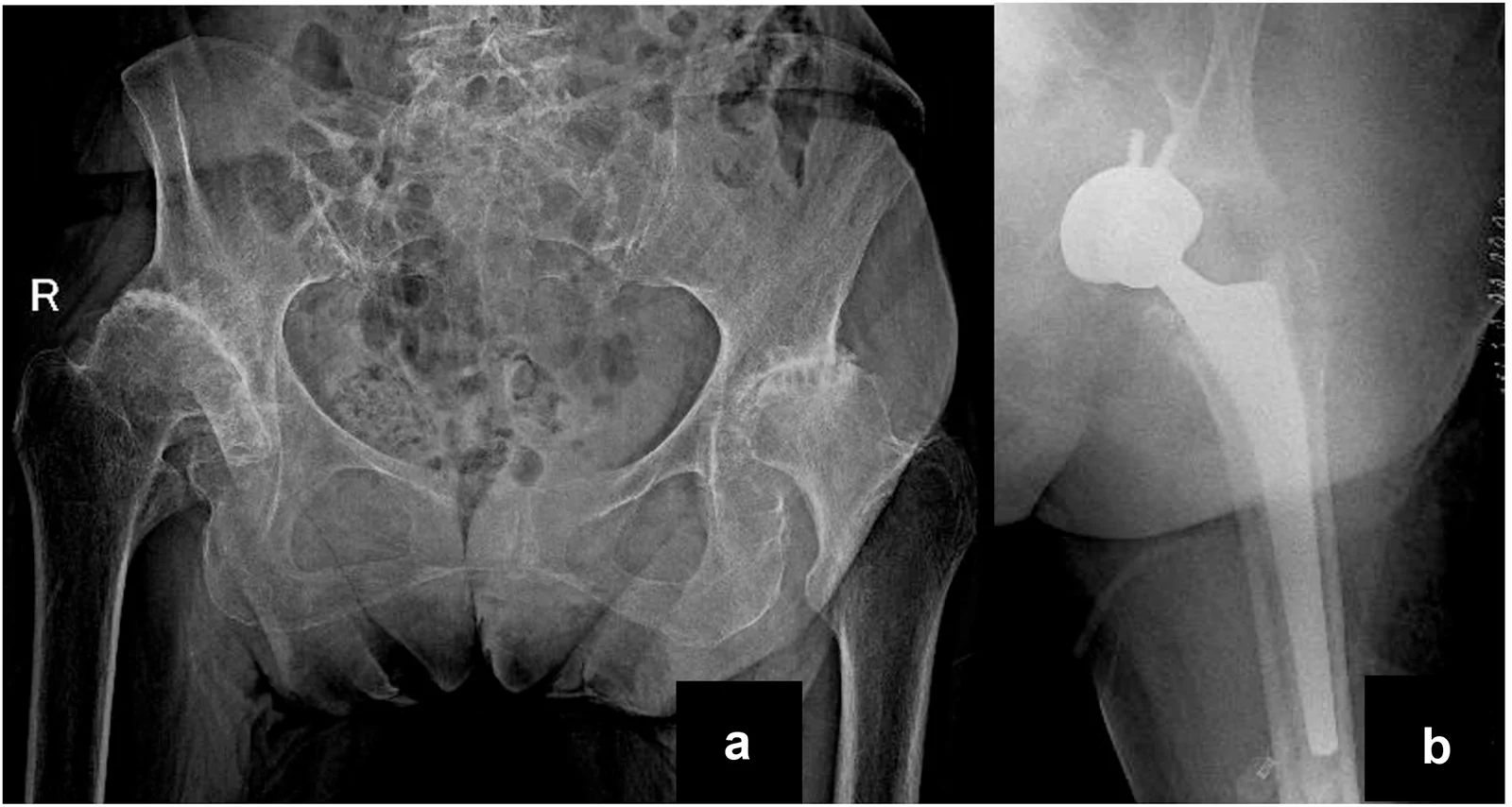
Preoperative pelvic radiograph showing severe bilateral hip osteoarthritis (a). First postoperative anteroposterior radiograph of the left hip showing early migration of the cup (b).

Her medical history included hypertension, dyslipidemia, diabetes, mild aortic stenosis, hypothyroidism, and chronic kidney disease, with a body mass index of 25. Relevant surgical history included a total hip arthroplasty, performed through a posterior approach, in another institution 2 weeks prior to presentation, for treatment of hip osteoarthritis (Fig. 1a). The index procedure was conducted 25 days before presentation by a senior surgeon with no robotics, computer navigation, or intraoperative imaging was used. The implants used were a press-fit metal back acetabular cup with three acetabular screws, a fixed polyethylene (PE) insert, a cemented Müller stem, and a metallic head. The patient returned home on day 3 postoperatively.

A radiograph performed at day 1 form the surgery was retrieved and had shown a protrusion of the cup with a fracture of the medial wall of the acetabulum, though the initial surgical report did not mention an intraoperative pelvic fracture. According to the explanation provided to the patient by her surgeon, no revision surgery was attempted because of her frail status, and a conservative treatment was first attempted by avoiding weight-bearing on the affected side (Fig. 1b). However, due to the increasing pain, the patient decided to seek another opinion.

Upon presentation in the outpatient clinic, the patient was using a wheelchair, as her symptoms prevented her from bearing weight on the left side, in addition to a notably shortened left lower extremity and swollen left thigh. Physical examination showed a closed wound with no redness or discharge. In addition to the functional incapacity of the affected limb, the patient presented severe reduction of the left hip range of motion (flexion: 40°, internal rotation: 5°, external rotation: 5°, abduction: 10°), probably due to the impingement of the greater trochanter on the pelvis secondary to the proximal migration. The patient did not report any episode of trauma following the first surgery. Neurovascular examination of lower limbs was strictly normal.

**Figure 2:**
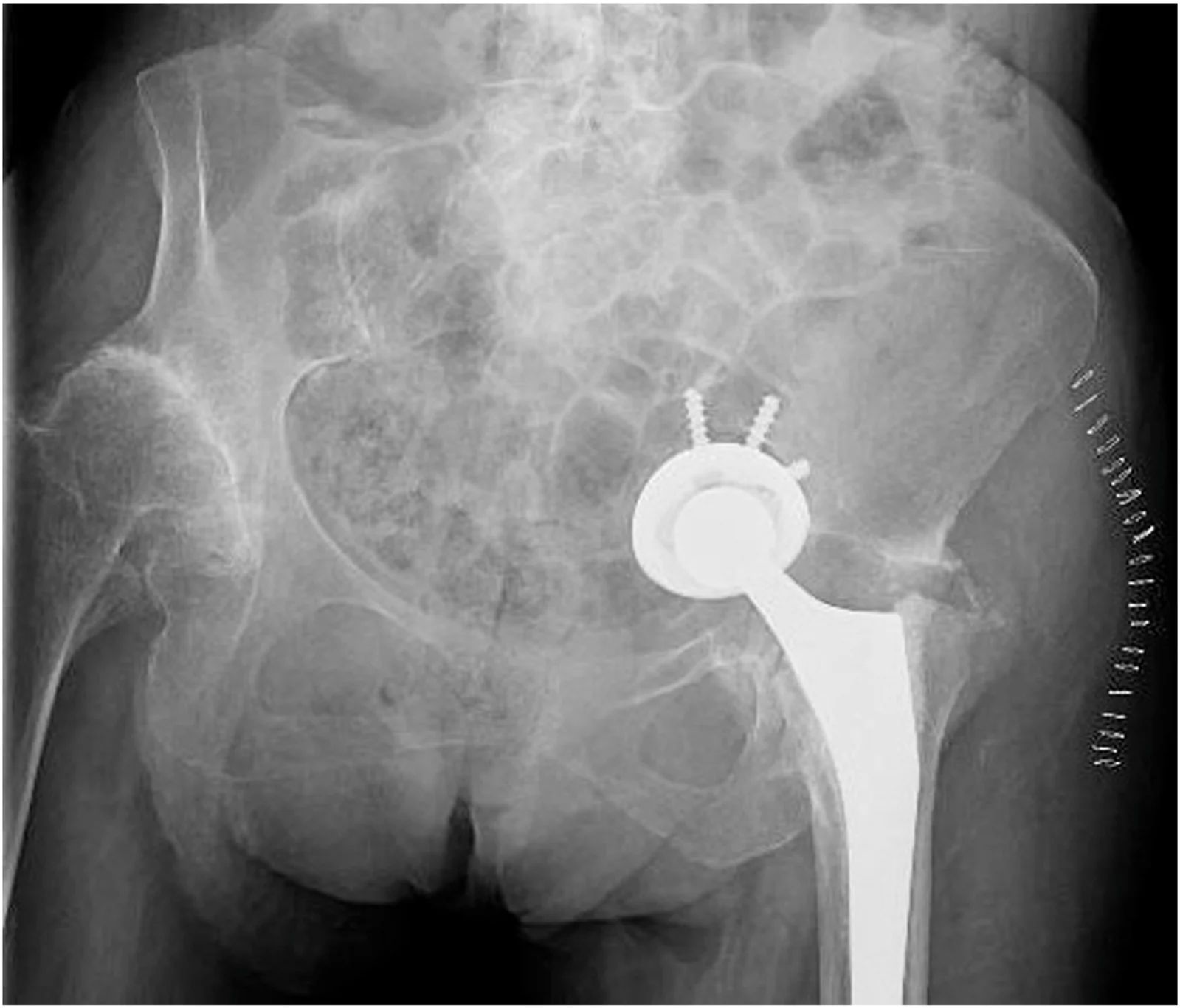
Anteroposterior pelvic radiograph upon presentation (progression of the intrapelvic cup migration).

**Figure 3:**
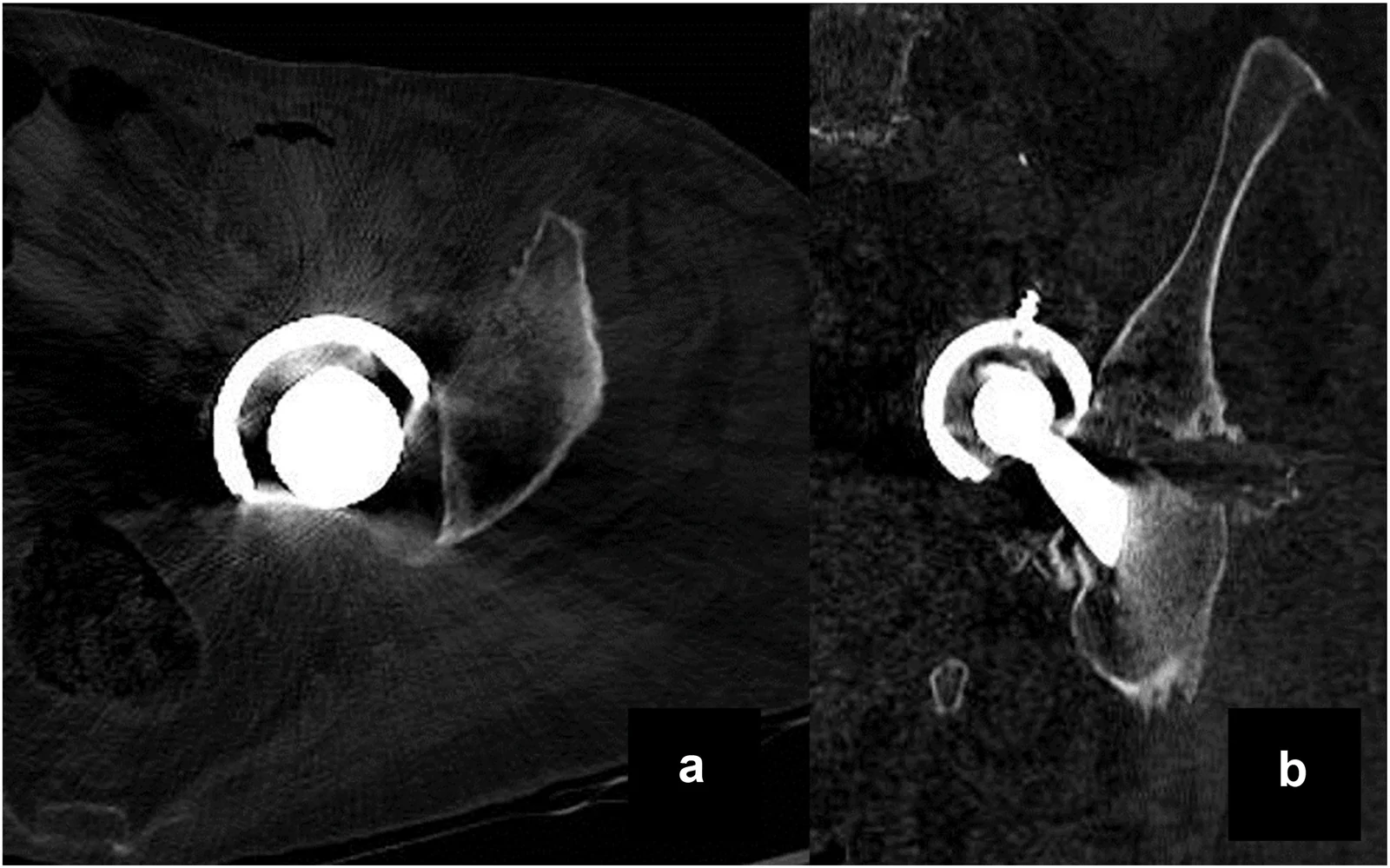
Intrapelvic cup migration. Axial (a) and coronal (b) tomodensitometric cuts.

An imagery workup was initiated during the consultation. New anteroposterior and lateral radiographs were taken, and showed a complete migration of the cup and its three divergent screws medially and proximally inside the pelvis (Fig. 2). Further investigations with a computerized tomography scan showed protrusion of the entire acetabular cup and screws superiorly and medially into the pelvis due to a fracture of the acetabular floor (Fig. 3) with complete destruction of the medial wall but preserved superior acetabular roof. No close proximity of gastrointestinal or urogenital structures was noted.

An angio-computerized tomography scan, performed in the same setting, revealed that two screws of the cup were abutting respectively the internal and external iliac veins without evidence of blood flow disruption or vessel aneurysm. No perforation of the urinary tract or digestive structures was noticed. Inflammatory blood markers’ values were within the normal range including white blood cell count (8500 WBC/microliter), erythrocyte sedimentation rate (11 mm/h), and C-reactive protein (3.8 mg/L), which was remarkable, considering these lab tests were performed at day 16 from index surgery. The patient was admitted the following day for surgery. The vascular surgeon was consulted and agreed to attend the first step of the surgery in case of any intraoperative vascular injury.

### First step: reconstruction of the acetabulum with bone grafting, insertion of a Kerboull cross-plate, and dual mobility cup

**Figure 4:**
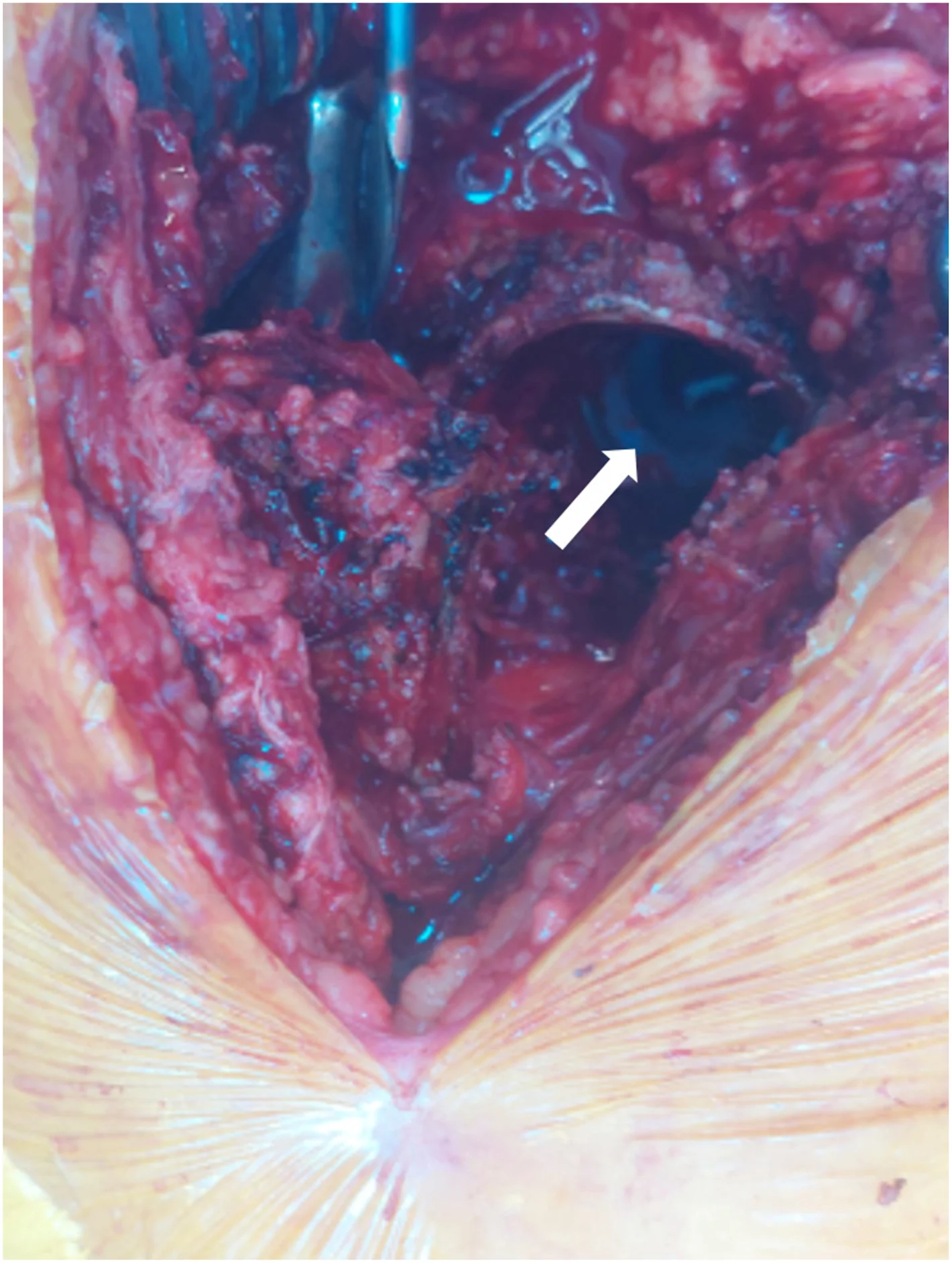
Intraoperative findings of the acetabular cup and PE (white arrow) seen through the acetabular defect.

Under general anesthesia, the patient was positioned in a lateral decubitus position with the left side up. Antibioprophylaxis with 2 grams of Cefazolin were given intravenously. Scrubbing with chlorhexidine and draping were performed in a sterile fashion. The same incision as the index surgery was used to expose the hip joint. Careful dislocation of the prosthetic hip was performed with traction using a Lambotte bone hook, with care being taken to avoid pushing the migrated cup further inside the pelvis. The prosthetic head was removed, revealing the intrapelvic cup and polyethylene liner that were seen through the acetabular defect (Fig. 4).

The initial plan was to remove the cup and reconstruct the acetabulum through the same incision, in a 1-stage surgery. However, many attempts to get hold of the polyethylene in order to remove it and get access to the screws’ heads were unsuccessful. Moreover, the soft tissue adherences, fibrous pseudomembrane formation, and increased bleeding risk associated with pulling the cup and screws led us to abandon this plan. Consequently, the decision was taken with the vascular surgeon participating in the case to leave the migrated cup inside the pelvis, reconstruct the acetabulum, and remove he first cup later with a second-stage surgery in order to avoid risks related with prolonged operating time and excessive bleeding. No loosening was noted concerning the prosthetic femoral stem; therefore, the decision was taken to keep it in place.

**Figure 5:**
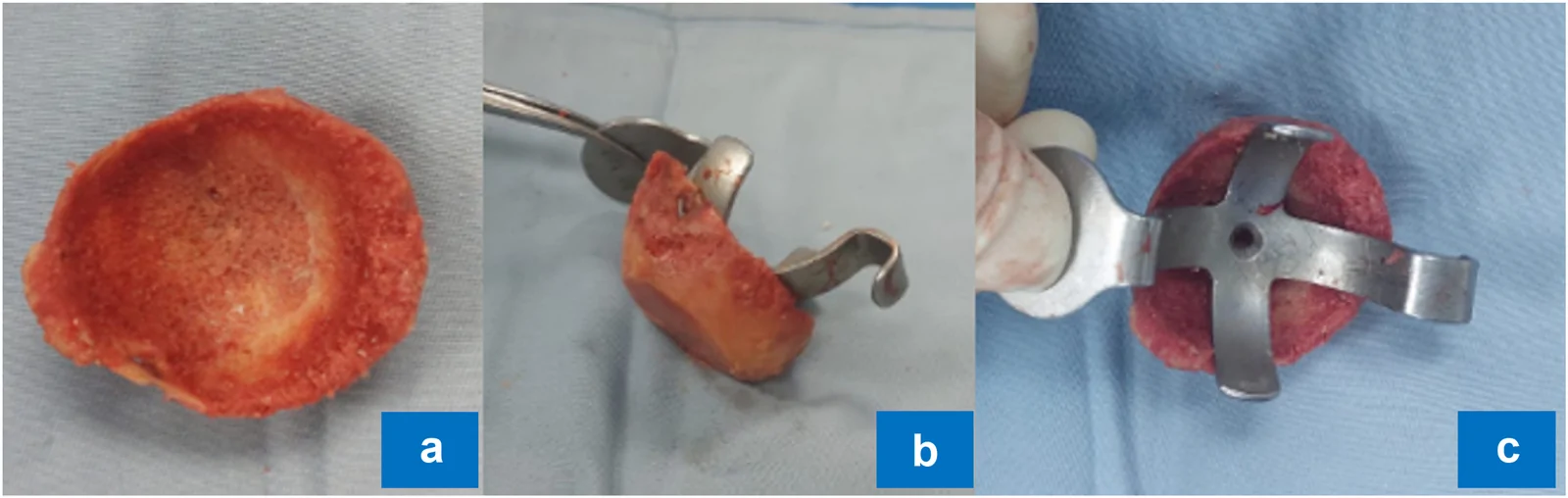
First step of acetabular reconstruction. Preparation of the femoral head allograft (a) to fill the acetabular bone defect and accommodate a Kerboull cross-cage (b and c).

**Figure 6:**
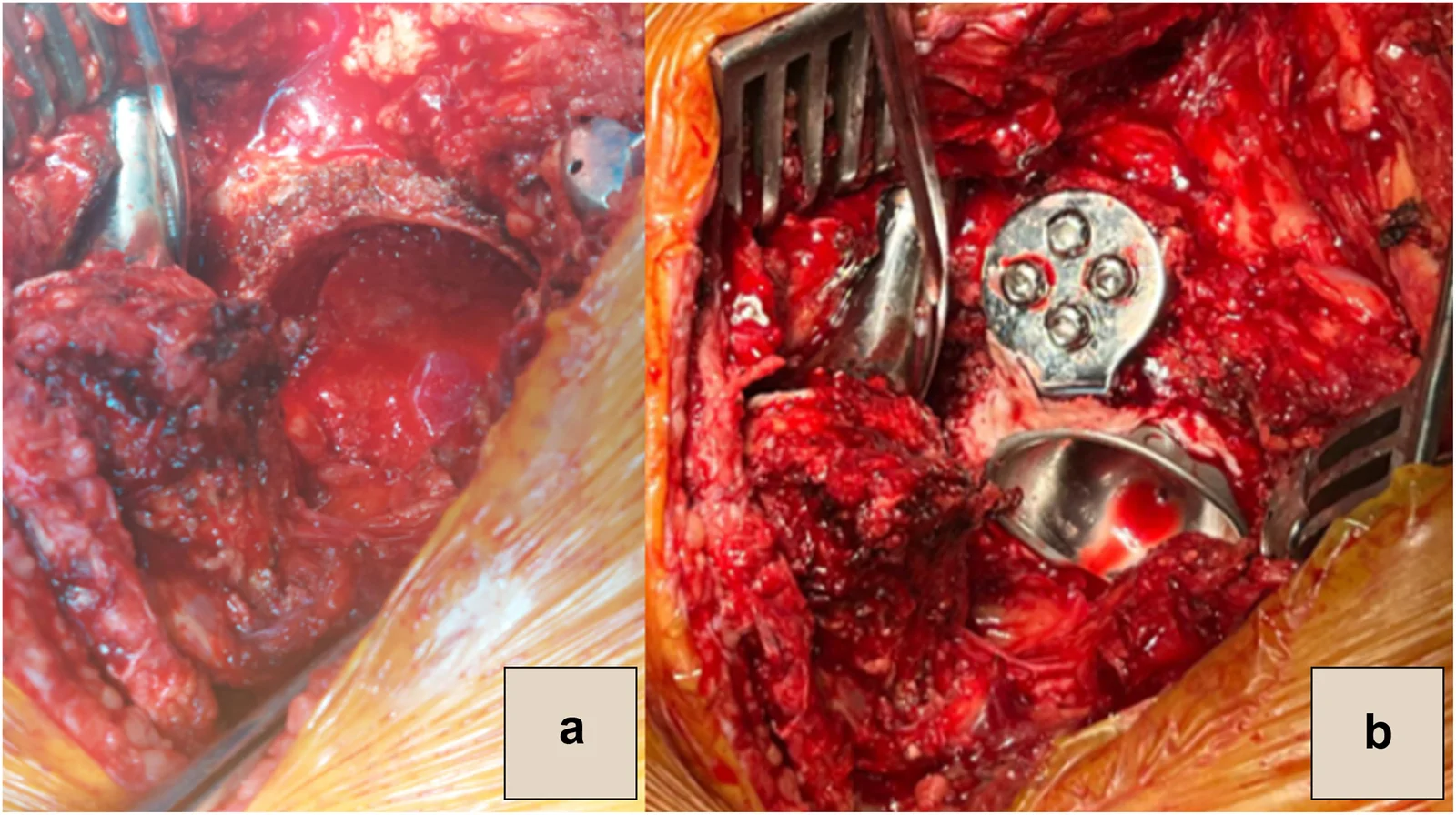
Second step of acetabular reconstruction. Acetabular aspect after impaction and reaming of the allograft (a), and after insertion of the Kerboull cross-plate and dual mobility cup (b).

The acetabular defect was addressed with bone graft reconstruction: after debridement of the nonviable bone, a femoral head allograft was sculpted using the acetabular reamer in order to obtain a shell-shaped bone block with a hemispheric concavity inside it (Fig. 5a). After confirming that the concavity could accommodate a Kerboull reinforcement cross-plate (Fig. 5b,c), the bone graft block was impacted to plug the medial wall defect (Fig. 6a). A Kerboull cross was then introduced using the previously described ‘’cross technique’’ [2] and its plate was fixed with four screws. A dual-mobility cup was then cemented inside the Kerboull cross in an adequate anteversion and inclination (Fig. 6b). Other options were available on the day of surgery, such as the Ganz reinforcement ring and the Burch–Schneider reinforcement cage. Surgeon preference went to the Kerboull cross-plate with which they were more familiar.

**Figure 7:**
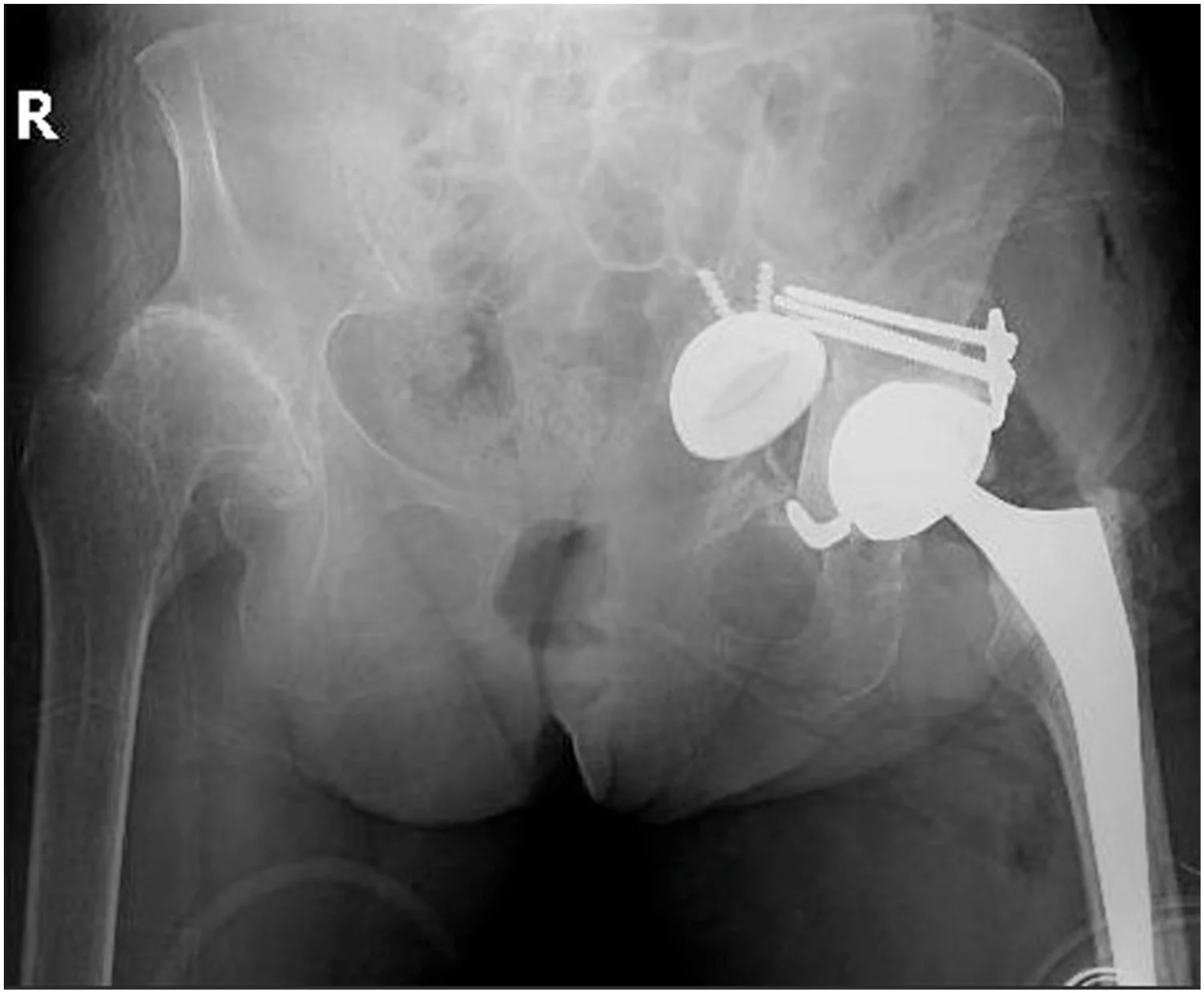
Postoperative pelvic radiograph showing the revised acetabular component and temporarily retained migrated intrapelvic cup.

Excellent stability was obtained. A ceramic head and polyethylene liner were inserted. Reduction of the hip joint was performed. Closure was done and patient was transferred to the recovery room in a stable status. Postoperative radiographs are shown in Figure 7. The patient was allowed to ambulate the very next day. Weight-bearing was allowed as tolerated and protected by a walking frame.

### Second step: removal of the migrated cup and screws through an anterior approach

After discussion with the vascular surgery team about the bleeding risks and the best approach to use, the patient was taken back to the operating room for removal of the intrapelvic cup that was left in place, through a retroperitoneal abdominal approach. The second surgery was originally planned to take place within 3 to 5 days. Unfortunately, the patient wished to be discharged to deal with family matters, and the procedure could only be scheduled 48 days later.

**Figure 8:**
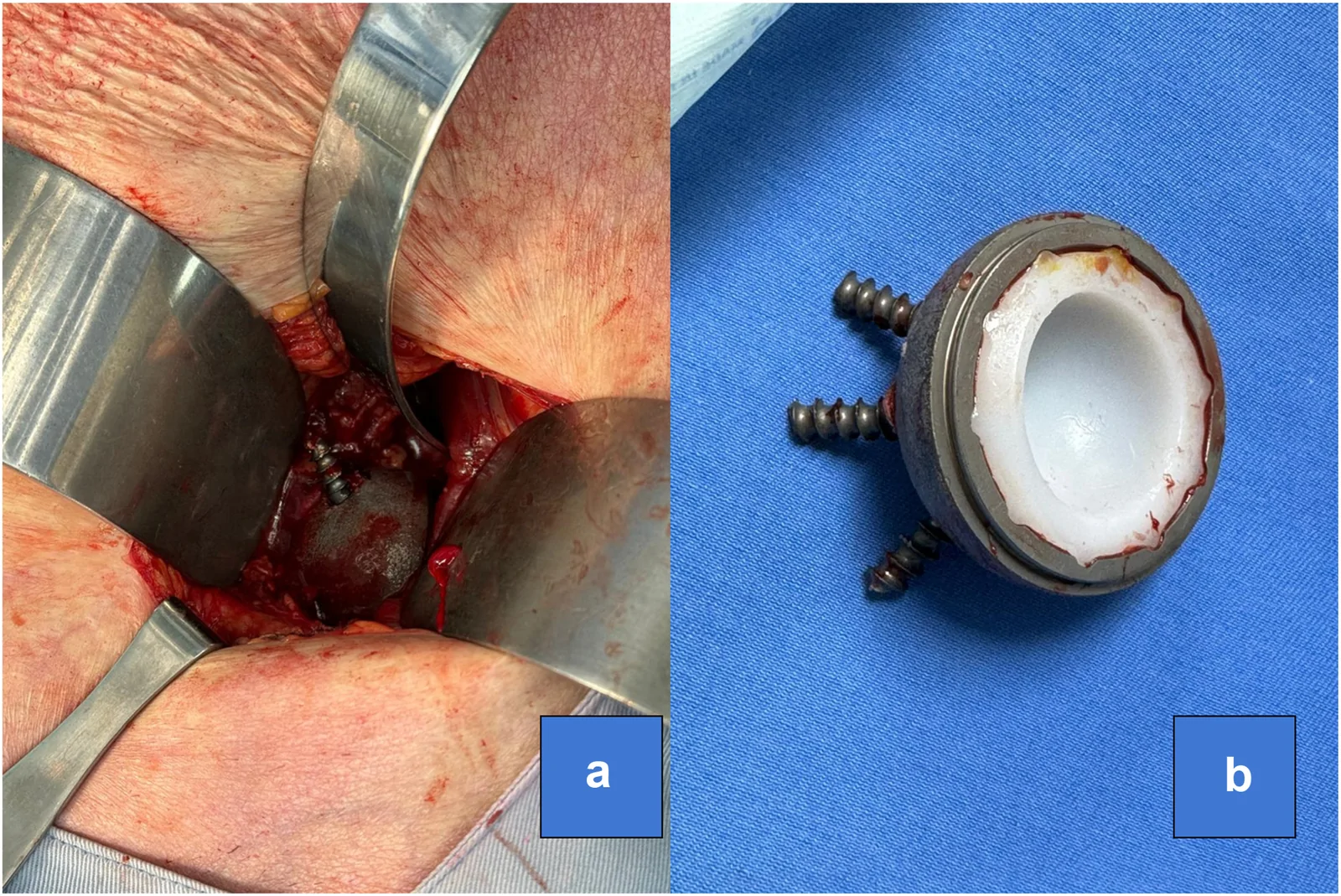
Second intraoperative images. The exposed acetabular cup and screws are seen after dissection through the abdominal incision (a). Details of the removed cup and sharp screws (b).

**Figure 9:**
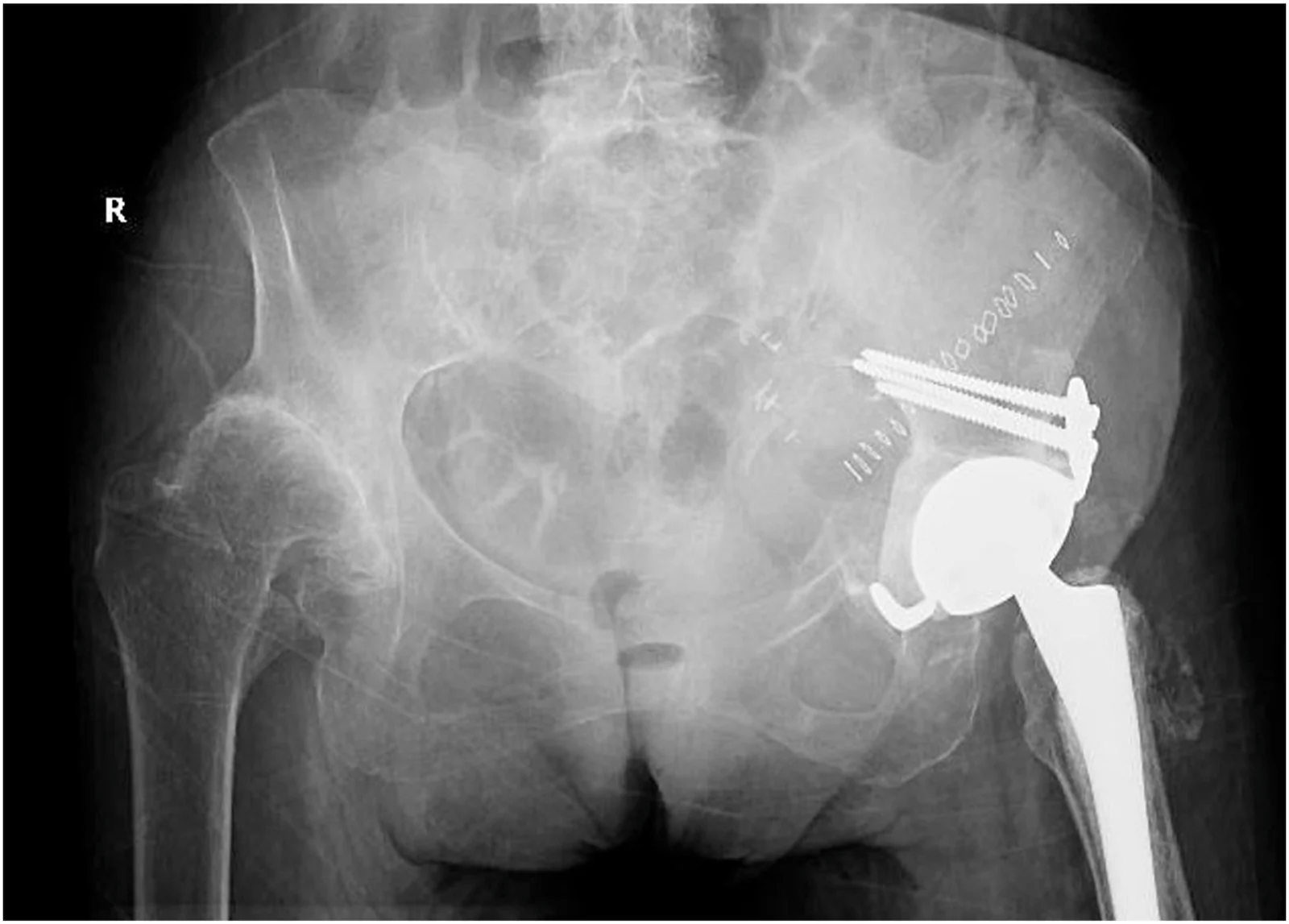
Final result. Anteroposterior pelvic radiographs after intrapelvic hardware removal.

Patient was put in a supine position on a regular table and an experienced vascular surgeon was scrubbed in to assist the orthopaedic surgeon operating on the case. After giving 2 grams of Cefazolin for antibioprophylaxis, a Rutherford–Morrison incision to the abdomen was performed, starting 2 cm above the anterior superior iliac spine, and extending obliquely down and medially. The retroperitoneal area was exposed through blunt dissection until exposure of the cup. All fibrous tissues were carefully dissected and separated from the metallic screws and the cup (Fig. 8), which were gently removed with no local complications (Fig. 9). Patient was discharged home on day 4 postoperatively and was able to walk with a cane or walking frame with no restriction regarding weight-bearing.

**Figure 10:**
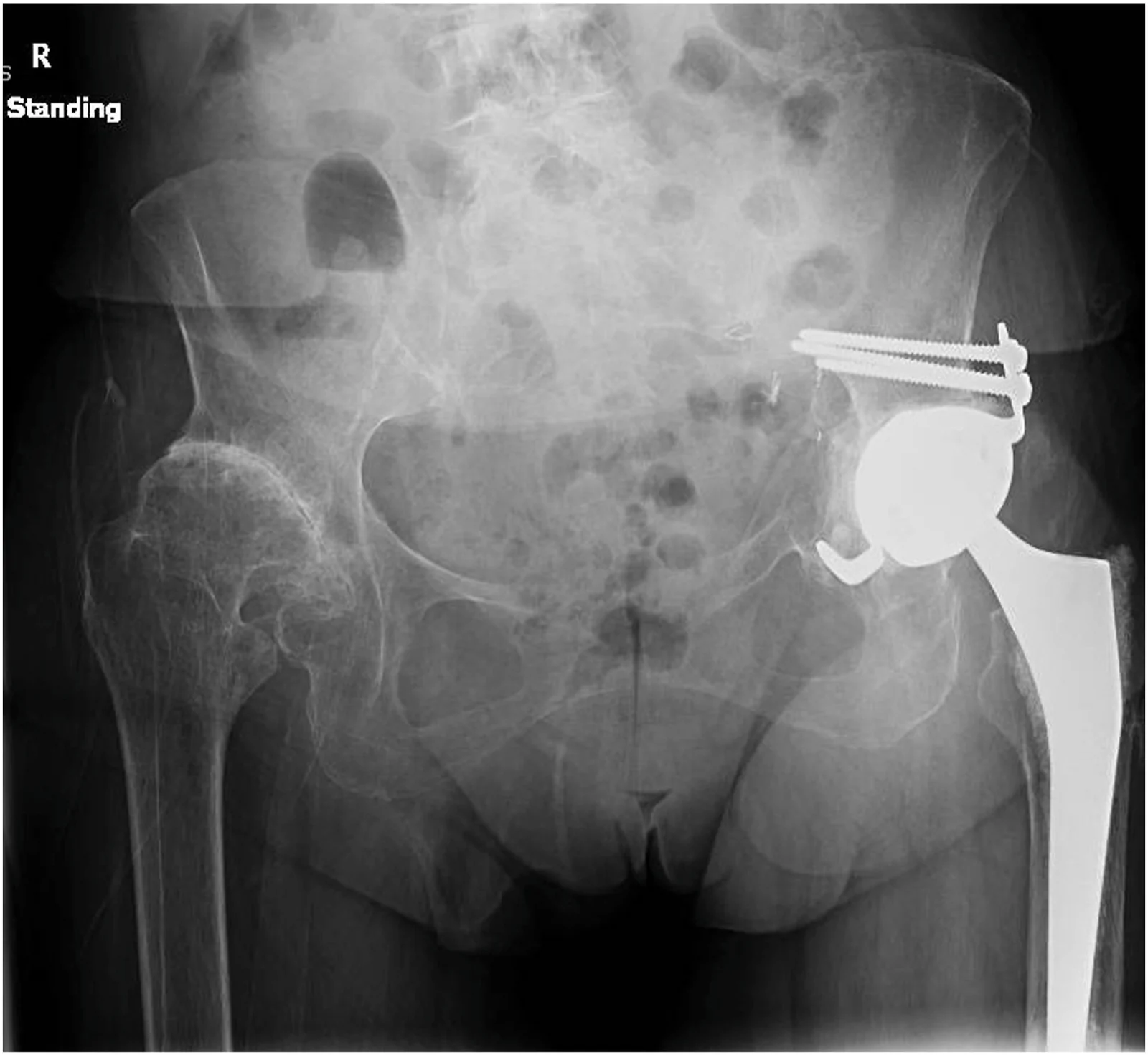
Radiographs 2.5 years postsurgery.

Follow-ups were performed at 1 month, 6 months, and 2.5 years (Fig. 10) postoperatively. At the last follow-up, the patient was able to ambulate without assistance and perform activities of daily living without pain. No local or general complications were recorded.

## Discussion

This case underscores the necessity for a customized surgical approach in managing intrapelvic cup migration, considering factors such as vascular, digestive, and urinary tract proximity, as well as soft tissue adherence. The effectiveness of addressing the acetabular defect with a one-block bone graft reconstruction and a Kerboull reinforcement plate, and removing the migrated cup through a delayed abdominal approach is highlighted in this unique sequence of events.

The absence of signs of infection, and the rapidity of the presentation following index surgery, coupled with the absence of trauma, suggest major flaws in the surgical technique of cup placement during the primary THA [1]. The most likely problem being a breach in the medial acetabular wall during reaming or cup impaction, which can substantially reduce the load-to-failure [3].

Some authors advocate a systematic two-approach attitude as soon as the cup migrates beyond the ilioinguinal line (indicating a displacement of the external iliac artery and veins, or the femoral nerve) [4]. For these reasons, but also because of the documented close proximity of the screws to important vascular structures, the dual approach strategy was preferred in this case. In fact, the presence of screws or pegs poses an additional risk factor for vascular injury by perforation or compression, as they can be more threatening than the cup itself [5]. This holds particularly true in the present case, since the used screws were long, with sharp threads, and sharper tips as they seemed to have been manually cut before insertion. Moreover, protrusion of the cup is usually accompanied by formation of a thick pseudo-membrane that requires careful dissection for safe removal of the cup [1,5]. These objectives cannot be attained only by operating through the medial acetabular wall defect using 1 single conventional hip approach.

When a dual approach strategy is deemed necessary, as in the present case, most authors prefer performing both approaches in 1 setting [[5], [6], [7], [8], [9], [10]], while only a minority prefers removing the failed implants first and delaying the THA revision surgery [[11], [12], [13]]. In either scenario, the migrated cup is systematically removed first, followed by revision of the acetabular component through a separate conventional hip approach. A recent literature review conducted by Papagianis et al. [14] documented only 17 cases of complete intrapelvic cup migration similar to the present case between 1993 and 2019. In four cases characterized by chronic migration and a fixed cup without vascular compromise, the authors advocate for THA revision while retaining the migrated cup within the pelvis to achieve a “wall-socket” construct, without the need for later removal [[14], [15], [16]]. However, keeping the failed implant in the pelvis definitively was not applicable to our case, as our patient presented a more acute migration and mobile intrapelvic hardware that posed a direct threat to intrapelvic vascularity.

Although the technique presented in this article shares similarities with the wall-socket technique, it distinguishes itself as the first reported case where the THA revision precedes the removal of the migrated intrapelvic cup, offering several advantages. First, dislocating a hip prosthesis under direct visual control is facilitated through a conventional hip approach. Second, it allows the patient to ambulate with weight-bearing in the event where the second surgery is delayed, as was the case here to avoid prolonged surgical time and potential increased blood loss. By revising the THA first, the need to place the patient in traction until the subsequent surgery to prevent soft tissue retraction was avoided. This strategy may therefore allow for better functional outcomes by avoiding muscle wasting. Last, performing acetabular wall reconstruction while the pseudo-membrane formed during cup migration is still present provides an additional safeguard against cement leakage into the pelvis and thermal injury to vital structures during polymerization.

It is crucial to recognize that while this strategy was successful in our case; its applicability may be limited in situations where infection is suspected. In such cases, where infected hardware must be removed and the infection eradicated prior to implantation of definitive revision hardware—whether the infection is the cause of failure or a result of intrapelvic organ involvement—our approach may not be suitable. It is noteworthy that nearly half the cases of acetabular socket protrusion reported in literature are associated with infection [4,17].

In the present case, the choice of device for acetabular reconstruction was made based on the algorithm proposed by Sporer et al. [18] in 2005, which selects the implant according to the severity of acetabular bone loss and the healing potential. Since the acetabular bone loss was close to causing a pelvic discontinuity—though with a potential for healing due to the acute and traumatic nature of the injury—a Kerboull plate with a cage was used in conjunction with a bone allograft to bridge the gap left by the protrusion and provide stable support for a new dual-mobility cup. Other implants, such as a triflange cup, would have been more appropriate if no healing potential remained, as in the case of massive chronic osteolysis.

Concerning the initial cup retrieval, Papagianis et al. [14] reported various surgical approaches, including Stoppa, paramedian, limited retroperitoneal, and pararectus techniques, selected based on surgeon preference and the extent of acetabular defect that needed to be addressed. Due to the paucity of such cases, no approach demonstrated unequivocal superiority over the others. The choice of surgical approach must therefore be tailored to each patient, with the surgeon possessing adequate familiarity and expertise in the selected technique. It is worth noting that some studies have highlighted the potential use of an arterial inflatable balloon, placed proximally to the affected iliac vessel, as an additional safety measure to control possible bleeding in the event of vascular injury [19,20].

## Summary

Early intrapelvic acetabular cup and screws migration, while rare, demands careful consideration and a customized surgical strategy. This case report sheds light on the challenges associated with early intrapelvic migration and provides valuable insights into an original successful intervention focusing on acetabular reconstruction first, followed by secondary removal of the retained intrapelvic implant.

**Key points**

• Prioritizing initial acetabular reconstruction followed by delayed cup removal is an effective strategy to mitigate vascular and visceral risks associated with the complex condition of early acetabular cup migration.
• Initial acetabular reconstruction provides technical advantages (such as an easier prosthesis dislocation through a conventional approach, or the opportunity to avoid cement leaking inside the pelvis).
• This strategy allows the patient to ambulate between the second surgery is the latter is to be delayed.
• Collaboration between surgical specialties is crucial for optimizing patient outcomes, and individualized surgical approaches should be guided by anatomical considerations, surgeon expertise, and the absence of infection.

## Conflicts of interest

The authors declare there are no conflicts of interest.

For full disclosure statements refer to https://doi.org/10.1016/j.artd.2026.102115.

## Informed patient consent

The author(s) confirm that written informed consent has been obtained from the involved patient(s) or if appropriate from the parent, guardian, power of attorney of the involved patient(s); and, they have given approval for this information to be published in this case report (series).

## CRediT authorship contribution statement

**Joe Ghanimeh:** Writing – review & editing, Writing – original draft, Visualization. **Kaissar Yammine:** Writing – review & editing, Validation, Methodology, Formal analysis. **Joeffroy Otayek:** Writing – review & editing, Resources, Data curation. **Anthony El Alam:** Writing – original draft, Visualization. **Fadi Hayek:** Supervision, Resources. **Camille Samaha:** Project administration, Conceptualization. **Chahine Assi:** Writing – review & editing, Project administration, Conceptualization.

## Appendix A Supplementary data

Conflict of Interest Statement for El Ala

Conflict of Interest Statement for Samaha

Conflict of Interest Statement for Assi

Conflict of Interest Statement for Hayek

Conflict of Interest Statement for Ghanimeh

Conflict of Interest Statement for Otayek

Conflict of Interest Statement for Yammine
